# Fluoxetine and Suicide Rates: Author's Reply

**DOI:** 10.1371/journal.pmed.0030504

**Published:** 2006-11-28

**Authors:** Julio Licinio

**Affiliations:** University of Miami, Miami, Florida, United States of America

Suicide is a complex outcome which cannot be attributed to a single factor. While we explored an association between suicide and antidepressant prescriptions, we fully acknowledge, as Camargo and Bloch suggest [1], that our work does not fully explain the observed trends. However, in other settings, the same association between increased antidepressant prescriptions and decreases in suicide have been observed. Please refer to the following article, published after ours came out in *PLoS Medicine*: “Increased antidepressant use and fewer suicides in Jamtland county, Sweden, after a primary care educational programme on the treatment of depression” [2]. I look forward to reading Camargo and Bloch's new article on the association of socioeconomic factors and suicide rates.

## References

[pmed-0030504-b001] Camargo CA, Bloch DA (2006). Fluoxetine and suicide rates: Suicide and the economy. PLoS Med.

[pmed-0030504-b002] Henriksson S, Isacsson G (2006). Increased antidepressant use and fewer suicides in Jamtland county, Sweden, after a primary care educational programme on the treatment of depression. Acta Psychiatr Scand.

